# Febuxostat ethanol monosolvate

**DOI:** 10.1107/S2056989020006076

**Published:** 2020-05-12

**Authors:** Thomas Gelbrich, Volker Kahlenberg, Verena Adamer, Sven Nerdinger, Ulrich J. Griesser

**Affiliations:** a University of Innsbruck, Institute of Pharmacy, Innrain 52, 6020 Innsbruck, Austria; b University of Innsbruck, Institute of Mineralogy and Petrography, Innrain 52, 6020 Innsbruck, Austria; c Sandoz GmbH, Biochemiestrasse 10, 6250 Kundl, Austria

**Keywords:** crystal structure, solvate, pharmaceuticals, hydrogen bonding, isostructural

## Abstract

The title compound, 2-(3-cyano-4-iso­but­oxyphen­yl)-4-methyl-1,3-thia­zole-5-car­b­oxy­lic acid ethanol monosolvate, C_16_H_16_N_2_O_3_S·C_2_H_6_O, (I), displays inter­molecular O—H⋯O and O—H⋯N bonds in which the carboxyl group of the febuxostat mol­ecule and the hydroxyl group of the ethanol mol­ecule serve as hydrogen-bond donor sites. These inter­actions result in a helical hydrogen-bonded chain structure. The title structure is isostructural with a previously reported methanol analogue.

## Abstract 2

Febuxostat and ethanol mol­ecules are linked into an O—H⋯O and O—H⋯N bonded chain structure.

## Chemical context   

Febuxostat is a novel, small-mol­ecule, non-purine-selective inhibitor of xanthine oxidase developed for the treatment of chronic gout and hyperuricemia, *via* oral administration (Pascual *et al.*, 2009 ▸; Gray & Walters-Smith, 2011 ▸; Kataoka *et al.*, 2015 ▸). This drug is currently marketed by Takeda Pharmaceuticals Inc. under the trade name Uloric. Matsumoto *et al.* (1999 ▸) disclosed the existence of five solid forms of febuxostat, *i.e.* of the anhydrous forms *A*, *B* and *C*, a methanol solvate *D* and a hemihydrate *G*. The crystal structures of two polymorphs were reported by Maddileti *et al.* (2013 ▸) and Yadav *et al.* (2017 ▸). Additionally, solvate structures containing the febuxostat mol­ecule and methanol (Jiang *et al.*, 2011 ▸), acetic acid (Wu *et al.*, 2015 ▸) or pyrdine (Zhu *et al.*, 2009 ▸) have been described.
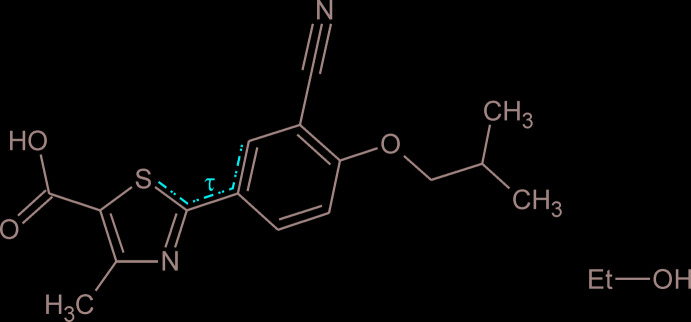


The current study was carried out as part of an investigation with the aim of establishing a modified synthetic route for febuxostat (Lutra *et al.*, 2012 ▸), avoiding harsh conditions, toxic reagents to form the thio­amide and the highly toxic cyanides. One of the key aspects of the novel route of synthesis was the introduction of a modified version of the Duff reaction (Duff & Bills, 1932 ▸, 1934 ▸) in the first step, which finally resulted in improved overall yields compared to the original synthesis by Hasegawa (1998 ▸).

## Structural commentary   

**Figure 1:**
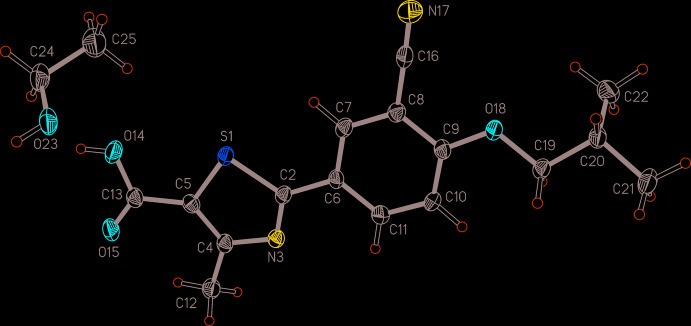
Asymmetric unit of (I) with displacement ellipsoids drawn at the 50% probability level and hydrogen atoms drawn as spheres of arbitrary size.

The febuxostat mol­ecule (Fig. 1 ▸) is essentially planar. This is illustrated by the fact that the mean plane defined by all its non-H atoms, except for C22 of the isobutyl group, results in a root-mean-square deviation for the 21 fitted atoms of only 0.0890 Å. Atom C22 is located at a distance of 1.498 (3) Å from this mean plane. All bond lengths and angles are in good agreement with the geometrical characteristics of previously determined febuxostat structures (see below). The relative mutual orientation of the CN substituent at the phenyl ring and the Me group at the thia­zole ring is characterized by the torsion angle S1—C2—C6—C7 of −6.5 (3)°. This torsion is also defined as τ in the Scheme. The isobut­oxy group adopts the expected extended chain geometry with C9—O18—C19—C20 = 175.3 (2)° and O18—C19—C20—C21 = 170.7 (2)°.

## Supra­molecular features   

**Figure 2:**
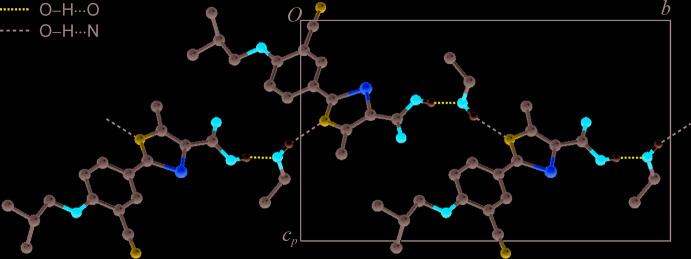
Hydrogen-bonded layer structure of (I), viewed along the *a* axis.

The carboxyl group of the febuxostat mol­ecule is linked to the OH group of an EtOH mol­ecule *via* an O23—H23⋯N3(−*x* + 1, *y* + 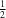, −*z* + 1) inter­action. The hy­droxy group of the solvent additionally serves as a hydrogen-bond donor group for an O14—H14⋯O23(*x* − 1, *y*, *z*) bond to a second febuxostat mol­ecule (see Table 1 ▸). Together, these two inter­actions result in a hydrogen-bonded chain composed of alternating febuxostat and ethanol mol­ecules that displays a 2_1_ symmetry and propagates parallel to the *b* axis (Fig. 2 ▸). The same hydrogen-bonded structure is also present in the analogous MeOH solvate of febuxostat, first reported (at 296 K) by Jiang *et al.* (2011 ▸) and redetermined by us at 173 K as part of this study (Gelbrich *et al.*, 2020*a* ▸). Indeed, a comparison with the program *XPac* (Gelbrich & Hursthouse, 2005 ▸) reveals that the EtOH and MeOH solvates are isostructural. The comparison of corresponding geometrical parameters generated from the complete set of 22 non-H atomic positions in the febuxostat mol­ecule resulted in a dissimilarity index (Gelbrich *et al.*, 2012 ▸) of *x* = 3.3, which indicates a high agreement of the febuxostat packing in the EtOH and MeOH solvates.

## Database survey   

Table 2 ▸ displays those entries in the Cambridge Structural Database (version 5.41, November 2019; Groom *et al.*, 2016 ▸) that relate to crystal structures containing the febuxostat mol­ecule. The febuxostat geometries in most of these structures are in good agreement with the parameters of (I), *i.e.* the torsion τ (see Scheme) typically adopts a value close to 0°. However, an opposite geometry with τ values close to 180° has been reported for the polymorphs Q and H1, a co-crystal with 4-amino­benzoic acid and a 2-(pyridin-2-yl­amino)­pyridinium salt.

## Synthesis and crystallization   

### Synthesis   

**Figure 3:**
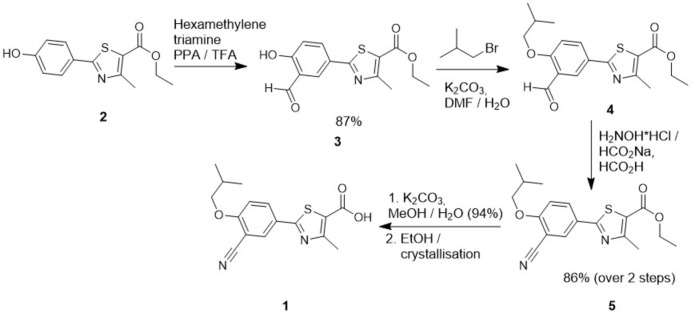
Synthetic scheme for the preparation of febuxostat (**1**).

The preparation of febuxostat was carried out according to the scheme in Fig. 3 ▸ in a modified procedure based on the original synthesis by Hasegawa (1998 ▸).

#### Ethyl 2-(3-formyl-4-hy­droxy­phen­yl)-4-methyl-5-thia­zole­carboxyl­ate (3)   

Ethyl 2-(4-hy­droxy­phen­yl)-4-methyl-5-thia­zole carboxyl­ate (**2**, 10.0 g) and hexa­methyl­ene­tetra­mine (5.86 g) were added to tri­fluoro­acetic acid (100 ml). The reaction mixture was heated to reflux under stirring for 40 h, and tri­fluoro­acetic acid was distilled out. The obtained residue was cooled to 298 K, water (200 ml) was added slowly, and the slurry was stirred for 4 h. After filtration, the product was washed and dried under vacuum to give 9.60 g of **3**.

#### Ethyl 2-(3-formyl-4-iso­but­oxy­phen­yl)-4-methyl-5-thia­zole­carboxyl­ate (4)   

Ethyl 2-(3-formyl-4-hy­droxy­phen­yl)-4-methyl-5-thia­zole­carboxyl­ate (**3**, 350 g), potassium carbonate (332 g) and isobutyl bromide (330 g) were added to DMF (1.75 1). The reaction mixture was heated to 383±3 K and stirred for 4 h. The reaction mixture was cooled to 298 K, and water (0.50 l) was added slowly. The slurry was stirred for 2 h. After filtration, the product was washed and dried under vacuum to give 389 g of **4**. ^1^H NMR (CDCl_3_), 400 MHz): *δ* = 1.079–1.101 (*d*, 6H), 1.366–1.413 (*t*, 3H), 2.185–2.230 (*m*, 1H), 2.769 (*s*, 3H), 3.914–3.935 (*d*, 2H), 4.316–4.387 (*q*, 2H), 7.045–7.074 (*d*, 1H), 8.188–8.225 (*dd*, 1H), 8.353–8.361 (*d*, 1H).

#### Ethyl 2-(3-cyano-4-iso­but­oxy­phen­yl)-4-methyl-5-thia­zole­carboxyl­ate (5)   

Ethyl 2-(3-formyl-4-iso­but­oxy­phen­yl)-4-methyl-5-thia­zole­carboxyl­ate (**4**, 350 g), sodium formate (123 g) and hydroxyl­amine hydro­chloride (84 g) were successively added to formic acid (1.4 l). The reaction mixture was heated to reflux and stirred for 5 h to complete the reaction. The reaction solution was cooled to 298 K, and water (2.8 l) was slowly added. After stirring for approximately 1 h, the slurry was filtered, the product was washed with water and dried under vacuum to give 321 g of **5**. ^1^H NMR (CDCl_3_), 400 MHz): *δ* = 1.053–1.104 (*d*, 6H), 1.368–1.463 (*t*, 3H), 2.164–2.225 (*m*, 1H), 2.768 (*s*, 3H), 3.890–3.911 (*d*, 2H), 4.324–4.395 (*q*, 2H), 6.998–7.027 (*d*, 1H), 8 8.188–8.225 (*dd*, 1H), 8.353–8.361 (*d*, 1H).

#### 2-(3-Cyano-4-iso­but­oxyphen­yl)-4-methyl-5-thia­zole carb­oxy­lic acid (1)   

Ethyl 2-(3-cyano-4-iso­but­oxy­phen­yl)-4-methyl-5-thia­zole­carboxyl­ate (**5**, 250 g) and potassium carbonate (200 g) were successively added to a mixture of MeOH (7.5 l) and water (250 ml). To complete the reaction, the solution was heated to reflux for 3 h under stirring. The clear solution was cooled, and vacuum was applied to distil out the solvent below 313 K. Water (5 l) was added to the residue. After stirring, EtOAc (2.5 l) was added. The solution was stirred, and the layers were separated. The pH of the aqueous solution was adjusted to 2.5±0.2 by adding diluted hydro­chloric acid solution at 313 K. After stirring for 1 h, the slurry was filtered, and the product was washed with water and dried under vacuum to give 215 g of **1**.

### Crystallization   

Febuxostat (1 g) was dissolved in ethanol (10 ml), which yielded a clear solution upon heating to 338 K. After filtration, the solution was allowed to cool to room temperature, and the subsequent crystallization resulted in febuxostat ethanol solvate.

## Refinement   

Crystal data, data collection and structure refinement details are summarized in Table 3 ▸. All H atoms were identified in difference maps. Methyl H atoms were idealized and included as rigid groups allowed to rotate but not tip (C—H = 0.98 Å), and their *U*_iso_ parameters were set to 1.5*U*_eq_(C) of the parent carbon atom. H atoms bonded to secondary CH_2_ (C—H = 0.99 Å) or tertiary CH (C—H = 0.99 Å) carbon atoms and H atoms bonded to C atoms in aromatic rings (C—H = 0.95 Å) were positioned geometrically and refined with *U*_iso_ set to 1.2*U*_eq_(C) of the parent carbon atom. H atoms in OH groups were identified in difference maps, refined with a distance restraint [O—H = 0.84 (2) Å] and a free *U*_iso_ parameter. Two outliers (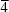 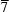
4) and (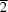,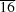,2) were omitted in the final cycles of refinement.

## Supplementary Material

Crystal structure: contains datablock(s) I. DOI: 10.1107/S2056989020006076/fy2145sup1.cif

Structure factors: contains datablock(s) I. DOI: 10.1107/S2056989020006076/fy2145Isup2.hkl

Supporting information file. DOI: 10.1107/S2056989020006076/fy2145Isup3.cml

CCDC reference: 2000973

Additional supporting information:  crystallographic information; 3D view; checkCIF report

## Figures and Tables

**Table 1:** Hydrogen-bond geometry (Å, °)

*D*—H⋯*A*	*D*—H	H⋯*A*	*D*⋯*A*	*D*—H⋯*A*
O23—H23⋯N3^i^	0.83 (2)	2.07 (2)	2.878 (3)	162 (4)
O14—H14⋯O23^ii^	0.84 (2)	1.80 (2)	2.631 (3)	170 (4)

Symmetry codes: (i) 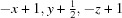; (ii) 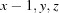.

**Table 2:** Conformation of febuxostat mol­ecules in polymorphs and multi-component structures, indicated by the torsion angle τ

Form	CSD	τ (°)	Ref.
Polymorph Q	HIQQAB	−174.1	Maddileti *et al.* (2013 ▸)
Polymorph H1	HIQQAB02	177.9	Yadav *et al.* (2017 ▸)
		−1.2	
MeOH solvate (173 K)	CCDC 1981184	5.6	Gelbrich *et al.* (2020*a* ▸)
MeOH solvate (296 K)	UREQOY	5.0	Jiang *et al.* (2011 ▸)
EtOH solvate (I)	–	4.5	This study
Acetic acid solvate (173 K)	CCDC 1981185	−2.8	Gelbrich *et al.* (2020*b* ▸)
Acetic acid solvate (296 K)	XULRUT	−3.2	Wu *et al.* (2015 ▸)
Pyridine solvate	PUHGUV	2.7	Zhu *et al.* (2009 ▸)
Acetamide co-crystal	HIQQEF	−6.9	Maddileti *et al.* (2013 ▸)
Nicotinamide co-crystal	HIQQIJ	0.7	Maddileti *et al.* (2013 ▸)
4-Amino­benzoic acid co-crystal	HIQQOP	−176.9	Maddileti *et al.* (2013 ▸)
Urea co-crystal	HIQQUV	4.4	Maddileti *et al.* (2013 ▸)
Isonicotinamide co-crystal	OYADAV	−3.8	Kang *et al.* (2017 ▸)
2-Methyl-1*H*-imidazole salt	FAMQIW	−19.4	Zhang & Zhang (2017 ▸)
		13.4	
Imidazole salt monohydrate	KIPMAA	−5.7	Gao *et al.* (2019 ▸)
2-(Pyridin-2-yl­amino)­pyridinium salt	FAMQOC	−174.5	Zhang & Zhang (2017 ▸)

**Table 3:** Experimental details

Crystal data	
Chemical formula	C_16_H_16_N_2_O_3_S·C_2_H_6_O
*M*_r_	362.43
Crystal system, space group	Monoclinic, *P*2_1_
Temperature (K)	173
*a*, *b*, *c* (Å)	4.7274 (2), 17.7820 (5), 10.7340 (4)
β (°)	98.994 (4)
*V* (Å^3^)	891.23 (6)
*Z*	2
Radiation type	Mo *K*α
μ (mm^−1^)	0.21
Crystal size (mm)	0.40 × 0.40 × 0.36

Data collection	
Diffractometer	Rigaku Oxford Diffraction Xcalibur, Ruby, Gemini ultra
Absorption correction	Multi-scan (*CrysAlis PRO*; Rigaku OD, 2015 ▸)
*T*_min_, *T*_max_	0.760, 1.000
No. of measured, independent and observed [*I* > 2σ(*I*)] reflections	6054, 3070, 2917
*R*_int_	0.028
(sin θ/λ)_max_ (Å^−1^)	0.641

Refinement	
*R*[*F* ^2^ > 2σ(*F* ^2^)], *wR*(*F* ^2^), *S*	0.030, 0.077, 1.04
No. of reflections	3070
No. of parameters	238
No. of restraints	3
H-atom treatment	H atoms treated by a mixture of independent and constrained refinement
Δρ_max_, Δρ_min_ (e Å^−3^)	0.26, −0.17
Absolute structure	Flack *x* determined using 1046 quotients [(*I* ^+^)-(*I* ^-^)]/[(*I* ^+^)+(*I* ^-^)] (Parsons *et al.*, 2013 ▸).
Absolute structure parameter	−0.02 (4)

Computer programs: *CrysAlis PRO* (Rigaku OD, 2015 ▸), *SHELXT* (Sheldrick, 2015*a* ▸), *SHELXL2014/6* (Sheldrick, 2015*b* ▸), *XP* in *SHELXTL* (Sheldrick, 2008 ▸), *Mercury* (Macrae *et al.*, 2020 ▸), *PLATON* (Spek, 2020 ▸) and *publCIF* (Westrip, 2010 ▸).

## supplementary crystallographic information

### Crystal data

**Table:**

C_16_H_16_N_2_O_3_S·C_2_H_6_O	*F*(000) = 384
*M**_r_* = 362.43	*D*_x_ = 1.351 Mg m^−^^3^
Monoclinic, *P*2_1_	Mo *K*α radiation, λ = 0.71073 Å
*a* = 4.7274 (2) Å	Cell parameters from 2681 reflections
*b* = 17.7820 (5) Å	θ = 2.3–28.6°
*c* = 10.7340 (4) Å	µ = 0.21 mm^−^^1^
β = 98.994 (4)°	*T* = 173 K
*V* = 891.23 (6) Å^3^	Prism, colourless
*Z* = 2	0.40 × 0.40 × 0.36 mm

### Data collection

**Table:**

Rigaku Oxford Diffraction Xcalibur, Ruby, Gemini ultra diffractometer	3070 independent reflections
Radiation source: fine-focus sealed X-ray tube, Enhance (Mo) X-ray Source	2917 reflections with *I* > 2σ(*I*)
Graphite monochromator	*R*_int_ = 0.028
Detector resolution: 10.3575 pixels mm^-1^	θ_max_ = 27.1°, θ_min_ = 1.9°
ω scans	*h* = −5→6
Absorption correction: multi-scan CrysAlisPro (Rigaku OD, 2015)	*k* = −17→22
*T*_min_ = 0.760, *T*_max_ = 1.000	*l* = −12→13
6054 measured reflections	

### Refinement

**Table:**

Refinement on *F*^2^	Secondary atom site location: difference Fourier map
Least-squares matrix: full	Hydrogen site location: mixed
*R*[*F*^2^ > 2σ(*F*^2^)] = 0.030	H atoms treated by a mixture of independent and constrained refinement
*wR*(*F*^2^) = 0.077	*w* = 1/[σ^2^(*F*_o_^2^) + (0.0439*P*)^2^ + 0.0696*P*] where *P* = (*F*_o_^2^ + 2*F*_c_^2^)/3
*S* = 1.04	(Δ/σ)_max_ < 0.001
3070 reflections	Δρ_max_ = 0.26 e Å^−^^3^
238 parameters	Δρ_min_ = −0.17 e Å^−^^3^
3 restraints	Absolute structure: Flack *x* determined using 1046 quotients [(*I*^+^)-(*I*^-^)]/[(*I*^+^)+(*I*^-^)] (Parsons *et al.*, 2013).
Primary atom site location: structure-invariant direct methods	Absolute structure parameter: −0.02 (4)

### Special details

**Table:**

Geometry. All esds (except the esd in the dihedral angle between two l.s. planes) are estimated using the full covariance matrix. The cell esds are taken into account individually in the estimation of esds in distances, angles and torsion angles; correlations between esds in cell parameters are only used when they are defined by crystal symmetry. An approximate (isotropic) treatment of cell esds is used for estimating esds involving l.s. planes.

### Fractional atomic coordinates and isotropic or equivalent isotropic displacement parameters (Å^2^)

**Table:**

	*x*	*y*	*z*	*U*_iso_*/*U*_eq_	
S1	0.32087 (12)	0.17683 (3)	0.31163 (5)	0.02221 (15)	
C2	0.3894 (5)	0.08540 (14)	0.3610 (2)	0.0195 (5)	
N3	0.2673 (4)	0.06576 (12)	0.45794 (18)	0.0199 (4)	
C4	0.1127 (5)	0.12385 (14)	0.4979 (2)	0.0210 (5)	
C5	0.1165 (5)	0.18918 (14)	0.4308 (2)	0.0209 (5)	
C6	0.5737 (5)	0.03525 (14)	0.3007 (2)	0.0188 (5)	
C7	0.6801 (5)	0.05673 (15)	0.1922 (2)	0.0211 (5)	
H7	0.6309	0.1045	0.1555	0.025*	
C8	0.8571 (5)	0.00904 (14)	0.1371 (2)	0.0208 (5)	
C9	0.9280 (5)	−0.06253 (15)	0.1882 (2)	0.0207 (5)	
C10	0.8252 (5)	−0.08344 (15)	0.2980 (2)	0.0231 (5)	
H10	0.8757	−0.1309	0.3357	0.028*	
C11	0.6499 (5)	−0.03524 (14)	0.3523 (2)	0.0225 (5)	
H11	0.5797	−0.0505	0.4266	0.027*	
C12	−0.0406 (5)	0.11107 (16)	0.6073 (2)	0.0269 (6)	
H12A	0.0985	0.1093	0.6852	0.040*	
H12B	−0.1448	0.0633	0.5962	0.040*	
H12C	−0.1761	0.1522	0.6125	0.040*	
C13	−0.0224 (5)	0.26111 (15)	0.4499 (2)	0.0234 (5)	
O14	0.0142 (5)	0.31184 (12)	0.36340 (19)	0.0339 (5)	
H14	−0.062 (8)	0.3537 (16)	0.375 (4)	0.072 (13)*	
O15	−0.1558 (4)	0.27251 (11)	0.53494 (18)	0.0345 (5)	
C16	0.9755 (6)	0.03373 (15)	0.0278 (2)	0.0259 (6)	
N17	1.0707 (5)	0.05481 (15)	−0.0569 (2)	0.0387 (6)	
O18	1.0943 (4)	−0.10584 (10)	0.12590 (16)	0.0248 (4)	
C19	1.1767 (5)	−0.17866 (15)	0.1797 (2)	0.0227 (5)	
H19A	1.0042	−0.2076	0.1919	0.027*	
H19B	1.2988	−0.1724	0.2627	0.027*	
C20	1.3396 (5)	−0.22023 (14)	0.0906 (2)	0.0236 (5)	
H20	1.4987	−0.1875	0.0709	0.028*	
C21	1.4684 (6)	−0.29130 (17)	0.1579 (3)	0.0327 (6)	
H21A	1.5817	−0.2775	0.2390	0.049*	
H21B	1.5919	−0.3164	0.1054	0.049*	
H21C	1.3139	−0.3254	0.1722	0.049*	
C22	1.1480 (7)	−0.24066 (17)	−0.0320 (3)	0.0336 (6)	
H22A	1.0040	−0.2773	−0.0148	0.050*	
H22B	1.2643	−0.2625	−0.0907	0.050*	
H22C	1.0519	−0.1953	−0.0694	0.050*	
O23	0.7299 (4)	0.43799 (11)	0.37730 (18)	0.0347 (5)	
H23	0.741 (8)	0.4681 (18)	0.437 (3)	0.053 (11)*	
C24	0.4973 (6)	0.45692 (16)	0.2805 (3)	0.0347 (7)	
H24A	0.5205	0.5091	0.2515	0.042*	
H24B	0.3143	0.4538	0.3142	0.042*	
C25	0.4914 (7)	0.40395 (19)	0.1727 (3)	0.0440 (8)	
H25A	0.4835	0.3521	0.2031	0.066*	
H25B	0.6647	0.4107	0.1343	0.066*	
H25C	0.3222	0.4141	0.1097	0.066*	

### Atomic displacement parameters (Å^2^)

**Table:**

	*U*^11^	*U*^22^	*U*^33^	*U*^12^	*U*^13^	*U*^23^
S1	0.0281 (3)	0.0153 (3)	0.0243 (3)	0.0024 (3)	0.0077 (2)	0.0010 (3)
C2	0.0205 (11)	0.0154 (12)	0.0214 (11)	−0.0005 (9)	−0.0003 (10)	−0.0001 (10)
N3	0.0210 (10)	0.0168 (11)	0.0219 (10)	−0.0017 (8)	0.0028 (8)	−0.0008 (8)
C4	0.0212 (12)	0.0173 (12)	0.0234 (12)	−0.0013 (10)	−0.0002 (10)	−0.0031 (10)
C5	0.0207 (11)	0.0209 (14)	0.0211 (11)	−0.0028 (10)	0.0034 (9)	−0.0034 (10)
C6	0.0199 (11)	0.0149 (12)	0.0211 (11)	0.0003 (10)	0.0014 (9)	−0.0033 (9)
C7	0.0237 (12)	0.0146 (12)	0.0244 (12)	−0.0014 (10)	0.0018 (10)	0.0006 (10)
C8	0.0231 (12)	0.0164 (12)	0.0225 (12)	−0.0018 (10)	0.0025 (10)	−0.0008 (10)
C9	0.0233 (12)	0.0167 (12)	0.0223 (12)	−0.0004 (10)	0.0040 (10)	−0.0033 (10)
C10	0.0303 (14)	0.0151 (13)	0.0240 (12)	0.0027 (10)	0.0046 (10)	0.0029 (10)
C11	0.0259 (13)	0.0200 (13)	0.0219 (12)	0.0001 (11)	0.0045 (10)	−0.0006 (10)
C12	0.0309 (14)	0.0218 (14)	0.0296 (13)	0.0002 (11)	0.0095 (11)	−0.0001 (11)
C13	0.0255 (12)	0.0192 (13)	0.0245 (13)	−0.0024 (11)	0.0006 (11)	−0.0046 (10)
O14	0.0474 (12)	0.0196 (10)	0.0387 (11)	0.0099 (9)	0.0189 (9)	0.0045 (9)
O15	0.0518 (12)	0.0232 (10)	0.0327 (10)	0.0046 (9)	0.0191 (9)	−0.0043 (8)
C16	0.0314 (13)	0.0166 (13)	0.0305 (14)	0.0045 (11)	0.0072 (12)	−0.0009 (11)
N17	0.0512 (15)	0.0318 (15)	0.0377 (13)	0.0033 (12)	0.0210 (12)	0.0081 (12)
O18	0.0328 (9)	0.0175 (9)	0.0260 (9)	0.0062 (8)	0.0105 (8)	0.0031 (7)
C19	0.0267 (13)	0.0162 (13)	0.0259 (12)	0.0018 (10)	0.0068 (10)	0.0024 (10)
C20	0.0264 (12)	0.0172 (13)	0.0292 (13)	0.0025 (10)	0.0108 (11)	0.0018 (10)
C21	0.0333 (14)	0.0225 (14)	0.0452 (16)	0.0077 (12)	0.0146 (13)	0.0072 (13)
C22	0.0447 (16)	0.0284 (16)	0.0290 (14)	−0.0006 (13)	0.0099 (12)	−0.0043 (12)
O23	0.0465 (12)	0.0218 (10)	0.0345 (11)	0.0092 (9)	0.0025 (9)	−0.0071 (9)
C24	0.0395 (16)	0.0237 (15)	0.0416 (16)	0.0086 (13)	0.0078 (13)	−0.0036 (12)
C25	0.0536 (19)	0.0343 (18)	0.0424 (17)	0.0044 (15)	0.0020 (15)	−0.0067 (14)

### Geometric parameters (Å, º)

**Table:**

S1—C2	1.725 (3)	O14—H14	0.84 (2)
S1—C5	1.733 (2)	C16—N17	1.139 (3)
C2—N3	1.313 (3)	O18—C19	1.446 (3)
C2—C6	1.466 (3)	C19—C20	1.511 (3)
N3—C4	1.372 (3)	C19—H19A	0.9900
C4—C5	1.369 (4)	C19—H19B	0.9900
C4—C12	1.491 (3)	C20—C22	1.520 (4)
C5—C13	1.467 (4)	C20—C21	1.534 (4)
C6—C7	1.393 (3)	C20—H20	1.0000
C6—C11	1.395 (3)	C21—H21A	0.9800
C7—C8	1.387 (3)	C21—H21B	0.9800
C7—H7	0.9500	C21—H21C	0.9800
C8—C9	1.406 (4)	C22—H22A	0.9800
C8—C16	1.444 (4)	C22—H22B	0.9800
C9—O18	1.350 (3)	C22—H22C	0.9800
C9—C10	1.394 (3)	O23—C24	1.430 (4)
C10—C11	1.383 (4)	O23—H23	0.83 (2)
C10—H10	0.9500	C24—C25	1.489 (4)
C11—H11	0.9500	C24—H24A	0.9900
C12—H12A	0.9800	C24—H24B	0.9900
C12—H12B	0.9800	C25—H25A	0.9800
C12—H12C	0.9800	C25—H25B	0.9800
C13—O15	1.205 (3)	C25—H25C	0.9800
C13—O14	1.325 (3)		
			
C2—S1—C5	89.55 (12)	N17—C16—C8	178.3 (3)
N3—C2—C6	123.6 (2)	C9—O18—C19	117.03 (18)
N3—C2—S1	114.17 (18)	O18—C19—C20	108.51 (19)
C6—C2—S1	122.25 (18)	O18—C19—H19A	110.0
C2—N3—C4	111.7 (2)	C20—C19—H19A	110.0
C5—C4—N3	115.0 (2)	O18—C19—H19B	110.0
C5—C4—C12	126.3 (2)	C20—C19—H19B	110.0
N3—C4—C12	118.7 (2)	H19A—C19—H19B	108.4
C4—C5—C13	128.6 (2)	C19—C20—C22	111.8 (2)
C4—C5—S1	109.62 (19)	C19—C20—C21	108.0 (2)
C13—C5—S1	121.80 (19)	C22—C20—C21	110.5 (2)
C7—C6—C11	118.3 (2)	C19—C20—H20	108.9
C7—C6—C2	121.3 (2)	C22—C20—H20	108.9
C11—C6—C2	120.4 (2)	C21—C20—H20	108.9
C8—C7—C6	120.7 (2)	C20—C21—H21A	109.5
C8—C7—H7	119.7	C20—C21—H21B	109.5
C6—C7—H7	119.7	H21A—C21—H21B	109.5
C7—C8—C9	120.6 (2)	C20—C21—H21C	109.5
C7—C8—C16	119.8 (2)	H21A—C21—H21C	109.5
C9—C8—C16	119.6 (2)	H21B—C21—H21C	109.5
O18—C9—C10	125.0 (2)	C20—C22—H22A	109.5
O18—C9—C8	116.4 (2)	C20—C22—H22B	109.5
C10—C9—C8	118.6 (2)	H22A—C22—H22B	109.5
C11—C10—C9	120.1 (2)	C20—C22—H22C	109.5
C11—C10—H10	119.9	H22A—C22—H22C	109.5
C9—C10—H10	119.9	H22B—C22—H22C	109.5
C10—C11—C6	121.6 (2)	C24—O23—H23	111 (3)
C10—C11—H11	119.2	O23—C24—C25	109.5 (2)
C6—C11—H11	119.2	O23—C24—H24A	109.8
C4—C12—H12A	109.5	C25—C24—H24A	109.8
C4—C12—H12B	109.5	O23—C24—H24B	109.8
H12A—C12—H12B	109.5	C25—C24—H24B	109.8
C4—C12—H12C	109.5	H24A—C24—H24B	108.2
H12A—C12—H12C	109.5	C24—C25—H25A	109.5
H12B—C12—H12C	109.5	C24—C25—H25B	109.5
O15—C13—O14	123.9 (2)	H25A—C25—H25B	109.5
O15—C13—C5	123.5 (2)	C24—C25—H25C	109.5
O14—C13—C5	112.7 (2)	H25A—C25—H25C	109.5
C13—O14—H14	113 (3)	H25B—C25—H25C	109.5

### Hydrogen-bond geometry (Å, º)

**Table:**

*D*—H···*A*	*D*—H	H···*A*	*D*···*A*	*D*—H···*A*
O23—H23···N3^i^	0.83 (2)	2.07 (2)	2.878 (3)	162 (4)
O14—H14···O23^ii^	0.84 (2)	1.80 (2)	2.631 (3)	170 (4)

Symmetry codes: (i) −*x*+1, *y*+1/2, −*z*+1; (ii) *x*−1, *y*, *z*.
